# The Spiral Curriculum: implications for online learning

**DOI:** 10.1186/1472-6920-7-52

**Published:** 2007-12-21

**Authors:** Kenneth Masters, Trevor Gibbs

**Affiliations:** 1 Faculty of Health Sciences, University of Cape Town, Cape Town, South Africa; 2 Bute Medical School, University of St Andrews, St Andrews, Fife, KY16 9TS, UK

## Abstract

**Background:**

There is an apparent disjuncture between the requirements of the medical spiral curriculum and the practice of replacing previous online material in undergraduate courses. This paper investigates the extent to which students revisit previous online material for the purposes of building the educational spiral, and the implications for the implementation of a Faculty's Learning Management System implementation.

**Methods:**

At the University of Cape Town, medical students' last date of access to 16 previous online courses was determined. Students completed a survey to determine their reasons for revisiting this material and the perceived benefits of this availability.

**Results:**

70% of the students revisited their previous online courses. The major reasons were to review lecture presentations, lectures notes, and quizzes. The perceived benefits were for understanding new material, preparation for assessments, and convenience.

Although student comments were not always in line with the concept of the spiral curriculum, most referred to processes of building on previous work, and some mentioned the spiral curriculum specifically.

**Conclusion:**

This study suggests that the practice of replacing previous online courses may hinder rather than support student learning. Although students visit previous material for ranges of reasons, a large number are aware of the spiral curriculum, and use the online environment to build upon previous material. Any practice, which entails replacing material and redesigning curricula content may be detrimental to the students' future learning needs, and such activities may need revision.

## Background

Electronic systems, also called Online Learning Management Systems (LMSs) or Virtual Learning Environments (VLEs), are used in higher education institutions to support face-to-face learning in a range of disciplines, including medicine [1,2]. They are also used to support "extra" student learning by providing an electronic resource area, and through organised course structures, such as cases, tutorials, modules, and student-years [3,4].

Understanding and maintaining the relationship between the face-to-face curriculum and the operation of the LMS is crucial; the face-to-face curriculum sets the agenda, teaching philosophy, and organisational structure, whilst the LMS is designed to support, expand and facilitate. Problems arise when common practices in LMS implementation hinder rather than support the face-to-face curriculum, and the needs of the face-to-face curriculum are no longer being met.

Much of the material in an LMS often consists of portable PowerPoint presentations and lecture notes, and a variable amount in other formats such as online quizzes and discussion board postings. Some of this material can be viewed only in the LMS itself, whilst other students and staff in response to queries and debates have contributed much of the material. This is simply a part of an online learning community [5,6]. For students, printing this material is not often a viable educational option – apart from negating the spirit of online learning, it is expensive, wasteful and difficult to store.

Related to technological and logistic needs, as students move from one year to the next, the standard practice can be to archive and replace previous online courses, with the view that students have now moved on to the next level of learning in their degree and have no need of the previous material. The implications of this practice for medical education will become clearer below.

### The medical spiral curriculum

Many modern medical training institutions have adapted Jerome Bruner's [7] concept of the "spiral curriculum," and use it in their medical teaching [8-13]. Also sometimes referred to as the "spiral of learning" [14] the spiral curriculum is based upon "an iterative revisiting of topics, subjects or themes throughout the course. A spiral curriculum is not simply the repetition of a topic taught. It requires also the deepening of it, with each successive encounter building on the previous one [10]."

Levels of difficulty and sophistication are increased, new learning is related to previous learning, and the students' competency is increased [10]. To provide added value, whilst maintaining the continuity in the spiral, "vertical themes" [2] or "golden threads" [15] are often developed throughout the curriculum.

The spiral is practiced in different ways at different institutions, but, essentially it recognises that courses in an undergraduate medical degree are components of a larger whole, and that, in addition to fitting together like pieces of a puzzle, they build up progressively, adding to student learning by building upon previously acquired knowledge. There is an assumption made, however that previously learned material is retained and built upon: the spiral is not solely a repository for revision material.

### The university of Cape Town

The University of Cape Town's (UCT) Faculty of Health Sciences currently uses an LMS (WebCT) to support its medical curriculum [16-18]. The medical degree is divided into 12 semesters across 6 years. The first 2 1/2 years follow the Problem-Based Learning (PBL) approach, while the rest of the degree resembles the more traditional clinical rotation years. In their first year, students also take a 2-semester multi-professional course [19].

The Faculty emphasises the concept of the spiral in the medical curriculum, and highlights it in documentation, aimed at both staff and students. For example, the Web site of Obstetrics and Gynaecology discusses the spiral curriculum [20], and the student handbook reinforces this by saying that the block "builds on the introduction provided in Semester 6 [3rd year] programme and forms part of a progressive spiral curriculum that runs through to the final year [21]." In addition, official curriculum documents discuss the use of the spiral [22], and educators in the faculty have described it in published papers [23]. There is also a great deal of informal reference to the spiral – some assessments make specific reference to the "spiral-based problem-based learning curriculum," and staff and students refer to the spiral in the LMS discussion boards. One such posting, by a student, complained that a section of the course had been trimmed, and, as a result, "spiral of learning won't develop fully."

### Previous studies

Probably because allowing students access to previous courses is not standard practice, no literature on the reasons for student usage of previous course material could be found. There are, however, some general figures and some evidence of institutional and students' difficulties when disengaging from their online course environment [24-27].

In a previous study [28], one of the authors (KM) examined medical students' access to previous courses, and found that an average of 69% of students had accessed the previous courses after they had officially ended, and that access continued for several years into the degree (albeit as low as 7% of the class). That study, however, did not tell anything about the students' activities in those courses, and whether or not the students were aware of their activities as participating in a spiral curriculum.

### The problem

There is little doubt that medical education is dynamic; new innovations lead to new course developments, varied teaching technologies and novel assessment procedures. Courses rarely stand still and frequently change year upon year. Although little is known of its degree, it is not unusual for course material to be replaced year upon year, often with the replacement of previous online courses in the LMS. How this then interacts or even interferes with the spiral curriculum is open to debate: the spiral's revisiting of previous material implies that students need to refer to previous work, but the previous work may no longer exist, often substituted by new material or material relevant only to the present student.

To determine the scope of the problem and attempt a solution, we need to answer two questions:

• Firstly, does the spiral curriculum really exist? While the spiral curriculum exists as an institutional policy and goal, and the curriculum map will tell us that the staff are revisiting and deepening the information, how do we know that the students are seeing and experiencing it as such, rather than merely seeing the later years as completely new and unrelated material? Is there a "hidden curriculum" [29] in which we believe that we have a successful approach, yet which is completely ignored by teaching staff and students, as "they find themselves unexpectedly trapped by grades (and grading), competition for success and the rewards that accompany it, and institutionalisation [29]." As Lowrie warns: "It is not enough merely to define the teaching content of a course. What teachers teach and what students learn may not be the same [30]." Reference has already been made to a student's posting regarding the spiral, but this might be an isolated incident. If the spiral exists in the majority of the students' minds, there would be ample evidence that they are actually revisiting previous materials, and for the purposes of developing the spiral as they approach new material later in their degree.

To answer this question, we aimed to determine the reasons for students' returning to the previous courses, and the benefits they perceived in having access to the previous courses. This information would be used to judge the students' awareness of the spiral curriculum and the value they attached to having the material available.

• Secondly, by implication, if the LMS is to support the spiral curriculum, what does this say of the practice of archiving and replacing previous courses in the LMS?

This paper attempts to answer the two questions by closely investigating student activities in courses that allow them to access previous materials in the LMS. The answers will have implications for other institutions that have spiral curricula and LMSs supporting their face-to-face activities.

## Methods

### Study 1

Study One repeated the measurement taken for the previous research [28], updating the number of examined courses from 13 to 16, and allowed the authors to judge whether the figures in the previous research were merely a statistical oddity or a trend.

Repeating the measurement also identified the student population size to be studied – targeting those students who *did *revisit their previous courses and determine their activities.

In keeping with the principles of the earlier research, students' last date of access to each course was recorded from the LMS logs. A calendar month was allowed after the official ending date of the online course. This allowed time for the new course to officially start, and ensured that all "official" activities, such as supplementary examinations, viewing of marks, etc., were complete. The last dates of access for each student to the courses were grouped into 6-monthly intervals.

### Study 2

Study 2, via a student questionnaire, was required to discover more about the student activities in the previous courses.

A starting point should have been a literature review of student activities in previous courses, to be used as a basis from which to develop a student questionnaire, but, as already been mentioned, no such studies could be found. Consequently, some 20 students in random pilot groups ranging across the degree were asked a single broad open-ended question: *"For what reasons do you go into old courses?"*

From these initial student responses, the possible reasons for accessing previous courses were to access:

1. PowerPoint presentations that staff created for the case-based material

2. Lecture notes that staff created for the case-based materials

3. Other material within the case environment (e.g. external links)

4. Other PowerPoint presentations

5. Anatomical Pathology quizzes (created in 2^nd ^year, this section runs almost exclusively as an online course)

6. Language of Medicine materials (this is a basic 2^nd^-year introductory sub-course)

7. Discussion board postings by staff, work related

8. Discussion board postings by students, work related

9. Discussion board postings, work related, with links and attachments

10. Discussion board postings – Off-course (i.e. "cafeteria") discussions

11. Casual browsing

12. Mistake (clicking on the course link in error)

The questionnaire design was based on these possibilities, and students were asked to rank these options in order of the frequency that each activity was a reason for their going into previous courses. The student rankings determined a weighting (1–12) assigned to each option. The mean of the ranking for each reason was calculated for each year and for the overall student body.

Because the initial random student groups might not have indicated all the possible reasons, the questionnaire allowed for students to add any other reasons. An open-ended question asked students to indicate the benefit they saw in having access to previous courses. In addition, the students were asked to identify the main benefits of having their previous courses available to them.

The data from the open-qualitative questions were coded into NUDIST, and then grouped into broad themes.

The survey specifically omitted all reference to the spiral curriculum, as it was felt that this might unduly influence the qualitative responses. (A question like "Do you know about the 'spiral of learning"' was considered to be too leading, and would result in responses that would over-state the students' knowledge of the spiral). The survey was delivered to 2^nd ^– 5^th ^year medical students in the Faculty, a total of 748 students. Although the survey was open to all students in the courses, the target group was specifically those students who revisited previous courses.

## Results

### Study 1

Table 1 below gives a breakdown of the figures showing the students' last date of access to their previous courses.

**Table 1:** Students' access to courses

**Course**	**N**	**Cut-off date**	**Percentage of students whose last date of access was**							
			
			**Within time**	**Months after cut-off date**						**Total after cut-off**
					
				**1–6**	**7–12**	**13–18**	**19–24**	**25–30**	**31–36**	
**4th-year courses (2 semesters)**										

C1	170	Jan-06	49	51						**51**

**3rd-year courses (1 semester)**										

C2	169	Jan-06	63	37						**37**
C3	168	Jan-05	39	27	17	17				**61**

**2nd and 3rd-year courses (3 semesters)**										

C4	217	July-05	29	24	47					**71**
C5	187	July-04	34	28	14	11	13			**66**

**1st- year courses (1 semester)**										

C6	236	Jan-06	22	78						**78**
C7	201	July-05	14	47	38					**86**
C8	349	July-05	12	50	38					**88**
C9	200	Jan-05	19	27	39	15				**81**
C10	353	Jan-05	67	16	8	9				**33**
C11	357	July-04	22	37	22	10	8			**78**
C12	200	July-04	14	36	18	15	19			**87**
C13	337	Jan-04	43	16	22	7	5	8		**57**
C14	200	Jan-04	15	18	27	22	12	8		**86**
C15	339	July-03	21	20	14	21	12	1	13	**79**
C16	195	July-03	24	28	13	11	11	6	7	**76**

Results, by course, total number of students (n), cut-off date, and percentage of students whose last date of access fell within official time, and in each 6-monthly interval after last official day of course

Apart from two courses (C2 and C10), every course shows more last accesses *after *the course cut-off than *within *the course time. Overall, a mean of 70%, a minimum of 33% and a maximum of 88% of students revisited their previous courses. Table 1 also demonstrates that large numbers of students are still revisiting their previous course material long after the course has officially ended.

Given that a mean of 70% of the students revisit previous courses, and the total student body is 748, this study also indicates that the target population size for Study 2 is 70% of 748 = 524.

### Study 2

223 (42%) of the students participated in the survey. Seven students stated that they did not use the previous courses at all, and one student did not answer any of the questions, so their records were discarded. This left a total of 215 valid responses. Therefore, our sample is 215/524 = 41.0%.reponse rate

Table 2 below lists the reasons, ranked according to the mean ranking score, where 1 = most important, and 12 = least important..

**Table 2:** Students' reasons for accessing previous courses

**Year:**	**2^nd ^Year**	**3^rd ^Year**	**4^th ^Year**	**5^th ^Year**	**Total**
**Reason/N**	**91**	**71**	**29**	**24**	**215**
**Cases-Power Points**	2.76	2.41	2.34	2.42	2.55
**Cases-Lecture Notes**	2.64	2.49	2.31	3.17	2.61
**Anatomical Pathology Quizzes**		3.76	5.45	7.00	4.77
**Cases-Other Material**	4.98	5.47	5.21	5.57	5.24
**Other Power Points**	5.86	4.93	5.14	5.41	5.41
**Disc. Boards Work Links and Attachments**	5.82	6.23	7.48	7.45	6.35
**Disc. Boards Staff, Work related**	6.09	6.78	8.81	8.45	6.94
**Lang of Medicine Materials**		7.09	7.00	8.38	7.30
**Disc. Boards Students, Work related**	6.93	8.58	8.85	9.41	7.99
**Casual Browsing**	9.60	9.89	9.54	8.82	9.60
**Disc. Boards Off-Course Discussions**	9.69	10.70	10.26	11.09	10.25
**By mistake**	10.38	11.02	10.00	10.00	10.50

In order of mean ranking, where 1 = most important and 12 = least important. (Note the exclusion of "Anatomical Pathology Quizzes" and "Language of Medicine Materials" from the 2^nd ^year course, as these are not "previous material" but are part of the 2^nd ^year course).

Whilst Table 2 allows us to see relative consistency across the years, there are variations, assumed to be specific student needs. The two reasons showing no re-visits in the year 2 column are second year courses only, with no material to revisit.

**Figure 1:**
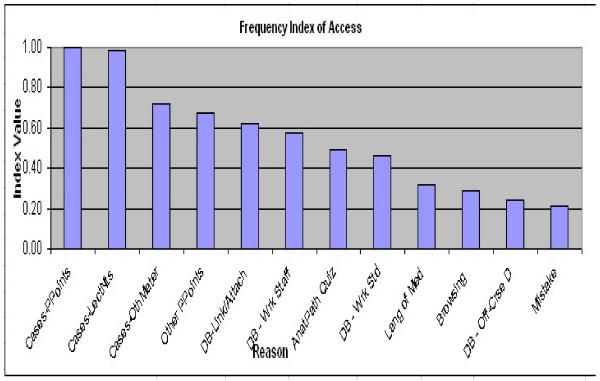
Graphical representation of the data.

**Figure 2:**
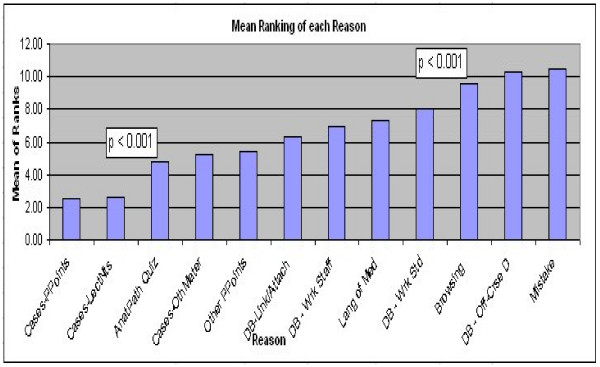
Graphical representation of the final column of mean rankings in Table 1, where 1 = most important and 12 = least important.

From a graphical representation of the data in Figure 1, there appears to be three clear groups of reasons (1–2; 3–9, 10–12), for students' revisiting previous material. These groupings are verified with a statistical analysis. A comparison of mean rakings for each item in 2-way comparisons, Figure 2, shows a statistically significant difference (p < 0.001) between "Cases – Lecture Notes" and "Anatomical Pathology Quizzes," and also a statistically significant difference (p < 0.001) between "Discussion Boards, Students, Work Related" and "Browsing". All other differences in the rankings are not statistically significant.

It is obvious that the lecture notes and PowerPoint presentations are the most important. Of the second group, the Anatomical Pathology Quizzes are the most important, and only work-related material is found in that second group. It is also obvious that casual browsing, off-course discussions and mistakenly entering the course play only a very minor role in the reasons for accessing the previous courses.

In response to "Other Reasons" for returning to courses, most students emphasised the accessing of lecture notes and presentations, while others spoke of administrative documents such as the plagiarism policy, past examination papers, private discussion boards (linked to PBL groups), old assignments, and to view old marks to judge progress,

When addressing the "Benefits of access" to previous courses, as would be expected from the statistics shown, 82% of the students spoke of the use of presentations and lectures for revision or reviewing purposes. There were three broad themes that emerged from the free texts allowed:

1) Revising old material for understanding of new material,

2) Revision for exams,

3) Convenience.

Examples of these are given below:

### Theme 1: Revising old material for understanding new material

Consistent with the quantitative data, most of the comments focused on the need to access previous lecture notes and presentations. There were nine explicit mentions of the "spiral of learning," while many others showed an awareness of the concept, sometimes using associated words found in the literature.

Some examples of comments are:

◦ *Need to go back and reread something in accordance with the whole spiral of learning!*

◦ *... also there are many gaps you realise from pre-clinical days and hence need to revise old notes*

◦ *Most of our work is a build up on material that we have already covered and the need to go over basic sciences is an absolute neccesity when it comes to the beginning of every block. Our cases also cover clinical aspects which were only theory when we were still doing pre-clinical work. Now these are relevant and practical so readily available access to this valuable resource is both time saving and allows for one to go back and fill the gaps they had in the past*.

◦ *In our spiral learning, we will constantly need to refer back to and consolidate prior knowledge with the new material. Having the lecture notes especially will ensure that this referring back process is swift and that we stick to the core, which can be found in the lectures!*

◦ *We can learn and reveiw old subjects that we dealt with but cannot remember well. Helps us to understand new material*.

◦ *As our convenor said, inside the new curriculum is the spiral of learning where you build upon what you have learnt from the previous year*.

◦ *Especially as you move ahead in your degree (MBChB), you constantly add on to the knowledge gained in your previous years. The course material that I encountered in my previous years provides a source of information that I can refer back to from time to time or whenever relevant*.

◦ *Lecturers often refer to old material learnt in previous years*

◦ *You are able to review old material, which you have covered. This could help rectify present gaps in knowldge and also reinforce information studied before*.

◦ *They are a quick reference and are very helpul... in the greater scheme of things, it seems that they encourage spiral learning*...

◦ *You can remind yourself on stuff that you have done previously, as our course is continous*.

◦ *They are concrete, easy to access evidence crucial to a lifetime learner as are doctors!*

Of interest is the multiple use of the word "revision," which might not be in the true spirit of the spiral curriculum. That said, however, it must be noted that this revision is not the result of students' looking at work done earlier in that year, but rather looking at work done a year or more before – they have recognised that, much of their new information will make little sense if not explicitly built upon work done in previous years. This is a major component of the spiral curriculum. A comment like "They are concrete, easy to access evidence crucial to a lifetime learner as are doctors!" demonstrates further that this spiral will continue into the years of medical practice.

### Theme 2: Revision, specifically aimed at tests and examinations

Naturally, the students are concerned with assessments, and several mentioned the value of revision in the context of these.

◦ *Mainly lecture notes that become relevant in later years or lectures that are repeated or the lecturer says "I did this lecture in 2nd year and expect you to remember it because it will be examinable!"*

◦ *Preparation for exams, revision purposes, referring back to old information*

◦ *You can find any old work that you need for future assessments*.

◦ *Revision; availability and access when needed; examination preparation*

### Theme 3: Convenience

Students also spoke about the convenience of accessing the material from the LMS rather than from their personal computers or paper records. This was usually because of ease of access, but also because of the quality of the material in its original format, and that the LMS serves as a backup if their own data are lost or destroyed.

◦ *The material covered is readily available and we don't have to waste time going through old files and textbooks. Also remind me as to what was core in that case*

◦ *To asssess the progress in our knowledge, building on what's we've done and have record of. It's also the safe and guaranteed way to keep past notes, lectures, comments, attachments and discussions, so it would be easy to go back to, during revision*.

◦ *They are permanently available even from home. Its faster to access than going to reopen old files and digging up old pieces of paper*

◦ *Easy access to material that I may not have downloaded or saved – material that is needed throughout medicine*

◦ *1) books are heavier 2) good refresh revision 3)they sometimes gets updated... thanks*

◦ *Accessible via the net anywhere I go. Not neccessary to download and keep vast documents on my very small space flash drive*.

◦ *To be able to access information that would otherwise cost a lot of money to print to keep as a hard copy*.

◦ *Well, mostly for the anat path tuts, where we can redo them and post them for marking, which cant be done in our homes*

◦ *... In addition, even the lectures I do have look way better in colour so it's more pleasant to access them via WebCT*. ....

◦ *WebCT quizes are also very valuable as they have colour pictures and not everyone is able to print all this out. Also, printing in colour is expensive at medschool and it is not as easy to see the pictures clearly if it is printed in black*.

### Other comments

There was the occasional perception of learning in context *("...also everything is in a case therefore in the context that it was meant"*), and the value in sharing information, formally or informally with students from other years ("*I'm an unofficial tutor for semester 1, 2 and 3 students and constantly need to revive and strengthen my knowledge of basic sciences and I use these old courses to keep myself in shape*.")

Of the seven students who indicated that they did not revisit their previous courses, only 1 offered a reason ("*I don't go into old courses, I save all the neccesary files onto my flash and store it on my home PC*.") Even this statement implies that the student is revisiting the previous material.

## Discussion

### Limitation of the study

A limitation of this study is the low response rate from the students, which is a little lower than found in many response rates of doctors [31,32]. Unfortunately, similar results have been experienced in other surveys of medical students [33-35]. Follow-up discussions with students revealed two major reasons for the low response rate in this research.

◦ Too many surveys – because the curriculum is new, the students complete numerous surveys and evaluations, and have reached a stage where their enthusiasm for these has waned. One student commented, *"Sometimes I think our main reason for being here is to supply information for staff research."*

◦ Clinical rotations in the later years are time-consuming and also do not afford students easy access to online computers. Because of this, when students do access their LMS, it is for their work, and surveys take a low priority.

A second limitation is that this study deals with a curriculum in one university only. There might be influencing factors that do not exist elsewhere.

### The role of the learning management system

The standard practice in the Faculty is to allow the medical students to *retain access to all their previous online courses as they progress through their degree*. The decision to implement this practice was taken by IT Education staff in conjunction with the teaching staff. It was done so in spite of the fact that we recognised that there would be logistical complications and difficulties arising from having multiple copies of courses currently available. The chief motivations for this decision were recognition of the:

◦ general immaturity of IT development in African students

◦ importance of the student-generated material in the courses,

◦ dictates of the curriculum to allow students to revisit previous material, most of which is in electronic form in the LMS.

The degree to which this process is mirrored by other institutions is not known.

The need for the electronic environment to support the face-to-face teaching environment for an outcome-based curriculum has been argued by Ross and Davies [36], and their solution was to build a database of materials based on the desired outcomes. While this solution is certainly better than no access at all, it requires further resources, and does not include the material contributed by students. The widespread use of LMSs means that, while not as effective as a true database, all the material is already in electronic format, searchable, and organised according to patterns familiar to the staff and students.

That the electronic environment should support rather than hinder the curriculum, however, still holds true, and a spiral curriculum in medical education requires an LMS to match its needs. While a curriculum map is a useful tool in this process, if the spiral is to be effective, students need to be aware of it, and need access to all their previous material.

The figures from Table 1, demonstrating the high rate of access after the course has officially ended, validate those of the previous research [28]. This, however, is only part of the investigation.

In the Introduction to this paper, two questions were asked. The first was *"Does the spiral of the curriculum really exist?*" Apart from a curriculum map, we wanted to know whether the students are aware of the concept, and are actively engaged in it. Our concern here was the extent to which the spiral was not part of the "hidden curriculum" experienced by students [29]. This is important: if the students are not aware and engaged, then they are merely attending a selection of medical courses, and the use of the LMS extends only as far as supporting the current course.

As mentioned above, the term "spiral curriculum" was specifically excluded from the questionnaire. In spite of this, the comments from the students in this study, and the obvious importance of access to previous lecture notes, presentations, quizzes and the like demonstrate that they are very aware of the spiral curriculum and engage directly with it. (Similarly, one notices that, while the word "gaps" is hardly jargon, it is a word used continually in the PBL process, and it is now part of the students' perception of the learning process.) An area of further research is a deeper exploration of the students' understanding of a spiral curriculum, its purpose and true educational value.

The second question was *"What does this say of the practice of archiving and replacing previous courses from the LMS?" *From this data, it is obvious that, if the LMS is to support the curriculum properly, it cannot simply be used on a year-by-year basis, with previous online material replaced and archived once students have finished with that course. The material that the students require for supporting the curriculum is in the LMS, and must be accessible, perhaps over an extended period.

One might argue that it is the students' responsibility to save all the materials they wish to have. As discussed earlier, however, this poses logistic problems for students, and much of the material, such as the quiz, is not in a "stand-alone" format. Besides, this expectation would be the equivalent of denying clinical students access to introductory library texts on the grounds that they should have made notes while they were studying in the earlier years.

### E-portfolios

One might argue that access to the LMS is not necessary if the university has an e-Portfolio system. While e-Portfolios are certainly valuable learning tools [37], their chief function is to keep a record of the student's own work, and for students to reflect upon their learning. For the e-Portfolio to have the LMS's functionality either the material must be duplicated in the e-Portfolio, dramatically increasing storage problems, or the e-Portfolio must link to the LMS material, requiring the LMS material to remain in existence anyway.

That said, however, an e-Portfolio tool *within *an LMS would provide the student with an ability to organise material according to personal preferences, while maintaining the original course structure.

### Outside medicine

Outside the field of medicine, other subjects, from physics and mathematics to literature and history, build on previous material and previous concepts. While they may not explicitly revisit material, they rely on knowledge of that material. Just as practising professionals need to refer to other sources, so the students, building up their knowledge, should have the facility to refer quickly and conveniently to previous, relevant material.

## Conclusion

This paper has argued and demonstrated that there is perhaps a disjuncture between the concept of the spiral curriculum and the standard practice of archiving and replacing previous online courses in an undergraduate medical curriculum. It has demonstrated further that, although students might not be fully conversant with the educational value of the spiral, they are at least somewhat *aware *of the spiral curriculum, and often return to previous online material for the purpose of building knowledge in the spiral.

It has pointed out, however, that maintaining such a system is not a trivial operation. There are questions of the length of courses' availability, and the difficulty and possible confusion of academic and support staff while working with so many concurrent multiple copies of courses.

Nevertheless, the practice of retaining previous courses is closely aligned with the philosophy of the spiral curriculum and delivers great benefits to the students, to the extent that it serves as a vindication of the spiral curriculum's success. Given this, these problems are worth solving, and the standard practice of archiving and replacing previous courses in a spiral curriculum, medical or otherwise, should be discussed and reviewed. As it stands, this study suggests that the practice of replacing previous online courses may hinder rather than support student learning.

### Practice Points

▪ The medical spiral curriculum requires students to revisit previous materials.

▪ Previous online courses are archived and replaced as students move through their degree.

▪ Given the opportunity, students in a spiral curriculum revisit previous online material specifically to build the spiral.

▪ The practice of archiving and replacing previous online courses in the degree must be reviewed.

## Competing interests

The author(s) declare that they have no competing interests.

## Authors' contributions

KM originally conceived the idea for the paper and wrote the initial draft.

TG reviewed the paper and added further ideas and comments.

Both authors have read and approved the final manuscript.

## Pre-publication history

The pre-publication history for this paper can be accessed here:


